# A case of hypophosphatemia and elevated intact fibroblast growth factor 23 levels after short-term saccharated ferric oxide administration in a young woman and database analysis of adverse drug reactions in Japan

**DOI:** 10.1016/j.bonr.2024.101754

**Published:** 2024-03-26

**Authors:** Teruhisa Kinoshita, Yuki Kondo, Yuka Sakazaki, Hiroki Imaizumi, Norio Takimoto, Yoichi Ishitsuka

**Affiliations:** a Department of Pharmacy, Kariya Toyota General Hospital, 5-15 Sumiyoshi-cho, Kariya, Aichi 448-8505, Japan; b Department of Clinical Chemistry and Informatics, Graduate School of Pharmaceutical Sciences, Kumamoto University, 5-1 Oehonmachi, Chuo-ku, Kumamoto 862-0973, Japan; c Department of Anesthesia, Kariya Toyota General Hospital, 5-15 Sumiyoshi-cho, Kariya, Aichi 448-8505, Japan

**Keywords:** Fibroblast growth factor 23, Saccharated ferric oxide, Hypophosphatemia

## Abstract

Intravenous iron replacement therapy is a common treatment for iron deficiency. Commonly used agents in this treatment include ferric carboxymaltose, ferric derisomaltose, and saccharated ferric oxide (SFO). These drugs are known to elevate fibroblast growth factor 23 levels, resulting in hypophosphatemia, but in past reports, hypophosphatemia attributable to SFO treatment has been associated mainly with prolonged administration over several weeks. The present study details our experience of a case of moderate hypophosphatemia (<2 mg/dL) in a 22-year-old woman who had no specific history of hypophosphatemia during the first 5 days of SFO treatment, and showed an increase in intact fibroblast growth factor 23 levels within the first week of treatment. Cases of hypophosphatemia have been reported as occurring as early as 1 week after the start of SFO administration in the Japanese Adverse Drug Event Report database. These cases, along with our case, underline the need for awareness of the possibility of hypophosphatemia from the early stage of SFO administration, regardless of the patient's age or dosage, as well as the need to monitor patients to prevent complications.

## Highlights

• Hypophosphatemia was seen within 1 week after initiation of saccharated ferric oxide.
• Intact fibroblast growth factor 23 was elevated accordingly.
• Several early-onset hypophosphatemia were also reported in the Japanese database.
• Serum phosphorus may decrease early after intravenous iron administration.
• Serum phosphorus levels should be monitored when using intravenous iron.

## 1 Introduction

The prevalence of iron deficiency, a primary cause of anemia, is estimated to exceed 15 % globally (Pasricha et al., 2021). Young women are especially prone to iron deficiency because of menstruation (Short and Domagalski, 2013), and they are treated with oral iron supplementation therapy for 3–6 months as iron replacement (Camaschella, 2015). However, it is often difficult to provide long-term oral iron replacement therapy because of side effects such as nausea, vomiting, and constipation, and thus, intravenous iron supplementation is used as an alternative therapy. In Japan, ferric carboxymaltose (FCM), ferric derisomaltose (FDI), and saccharated ferric oxide (SFO) are used in intravenous iron supplementation, but these drugs are known to cause hypophosphatemia associated with increased intact fibroblast growth factor 23 (iFGF23) levels (Edmonston and Wolf, 2020).

FCM and SFO are more likely to cause hypophosphatemia than FDI, and the frequency of hypophosphatemia varies according to the form of intravenous iron supplementation given (Wolf et al., 2020). The duration of hypophosphatemia also varies according to the type of iron given, and hypophosphatemia attributable to FCM is especially slow to improve (Wolf et al., 2020; Wolf et al., 2018; Hardy and Vandemergel, 2015). FCM and SFO have also been demonstrated to cause clinical complications such as osteomalacia and fractures (Vilaca et al., 2022), and clinicians should be vigilant for hypophosphatemia when intravenous iron is administered.

The onset of hypophosphatemia is reported to occur relatively early during FCM administration (Wolf et al., 2020). Furthermore, although SFO-induced hypophosphatemia is often associated with long-term administration, past reports have found that the peak onset of hypophosphatemia attributable to SFO was 2–6 weeks after the start of administration (Glaspy et al., 2021; Kawabata et al., 2022; Ikuta et al., 2019). However, there is little information regarding whether SFO-induced hypophosphatemia occurs within the first week of treatment.

In this study, we report a case of moderate hypophosphatemia (<2 mg/dL) occurring on the 5th day of treatment with SFO 80 mg/day in a 22-year-old woman with no underlying disease. The findings in this patient included elevated iFGF23 and intact parathyroid hormone (iPTH) levels and decreased blood 1,25-hydroxyvitamin D levels during the first week of treatment.

## 2 Case presentation

A 22-year-old woman with no specific medical history and/or comorbidities who was taking no regular medications was rushed to another hospital for seizures, but was discharged with no abnormal findings. Because her serum phosphorus levels were not measured at that time, it was unknown whether there were any abnormalities. Three days later, she had another seizure, and was admitted to our hospital (day 0). Anti-*N*-methyl-d-aspartate (NMDA) receptor encephalitis was suspected, and a spinal fluid examination was performed. Blood examination on admission revealed anemia with a hemoglobin level of 9.2 g/dL. The next day (day 1), computed tomography revealed a left ovarian teratoma that was suspected of being associated with anti-NMDA receptor encephalitis, and emergency laparoscopic partial left ovarian resection was performed. Blood examination revealed low serum iron (7 ng/mL) and ferritin levels (20 μg/dL), and thus, SFO 80 mg/day was started (Table 1). Plasma exchange therapy (albumin replacement) was given for anti-NMDA receptor encephalitis on days 3, 5, 7, 9, and 12, and steroid pulse therapy (methylprednisolone 1000 mg/day) was provided for 3 days starting on day 4.

**Table 1:** Relevant data at the start of intravenous iron administration.

Day 1		Normal range
Hemoglobin	8.8 g/dL	11.6–14.8 g/dL
Serum iron	20 μg/dL	40–188 μg/dL
Ferritin	7 ng/mL	8–74 ng/mL
Erythrocyte mean corpuscular volume	75 fl	83.6–98.2 fl
Erythrocyte mean corpuscular hemoglobin	23.2 pg	27.5–33.2 pg
Erythrocyte mean corpuscular hemoglobin concentration	30.9 g/dL	31.7–35.3 g/dL
Alkaline phosphatase	53 U/L	38–113 U/L
Serum calcium	9.0 mg/dL	8.8–10.5 mg/dL
Serum albumin	3.8 g/dL	4.1–5.1 mg/dL

Nutritional therapy consisted of amino acids, sugar, electrolytes, and vitamin B1 solution (420 kcal/day) starting on day 1, and Peptamen AF® (360 kcal/day) was added on day 3.

After laparoscopic partial left ovarian resection (day 1), the patient was admitted to the intensive care unit for systemic management with noradrenaline, remifentanil, thiamylal sodium, dexmedetomidine, and propofol. On day 2, the patient's serum phosphorus level was normal (4.3 mg/dL), but it decreased to 3.6 mg/dL on day 4 and 2.6 mg/dL on day 5. Therefore, phosphate (10–30 mmol as phosphorus) replacement was started. However, on day 6, her serum phosphorus level was 1.4 mg/dL, and it further decreased to 1.2 mg/dL on day 7. On day 8, vitamin D, iPTH, and fibroblast growth factor 23 (FGF23) levels were measured, and SFO administration was discontinued.

**Fig. 1:**
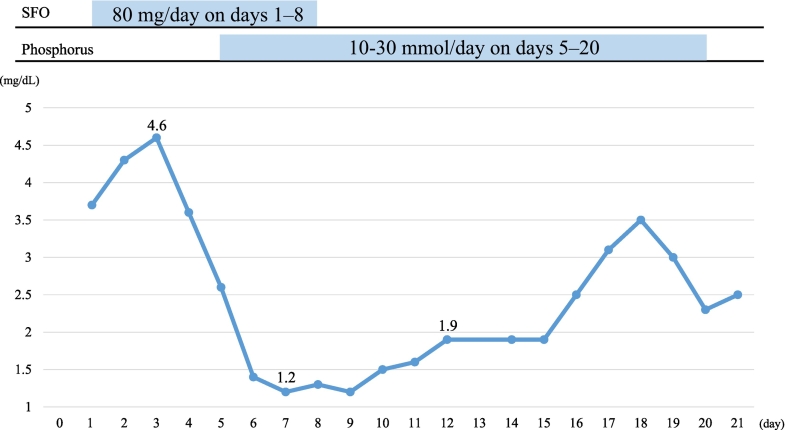
Serum phosphorus levels during hospitalization in the current patient.

Daily phosphate (20 mmol as phosphorus) supplementation was continued, but hypophosphatemia persisted. The patient's phosphate level did not exceed 2.5 mg/dL until day 16 (Fig. 1). On the same day, the patient displayed an abnormally high iFGF23 level of 209 pg/mL (Table 2).

**Table 2:** Serum phosphorus level-related marker levels in the patient on day 8.

Day 8		Normal range
25OH vitamin D	4 ng/mL	30 ≤ ng/mL
1.25(OH)2 vitamin D	8.1 pg/mL	20–60 pg/mL
iPTH	174 pg/mL	10–65 pg/mL
iFGF23	209 pg/mL	19.9–52.9 pg/mL

Treatment was continued and the patient survived. The patient was discharged from the intensive care unit (ICU) on day 28 after admission and discharged home on day 69.

## 3 Discussion

FGF23, which is secreted primarily by osteocytes, serves as a regulatory hormone for blood phosphorus levels. FGF23 acts on the proximal tubules of the kidneys to inhibit phosphorus reabsorption and reduce blood 1,25-hydroxyvitamin D levels, thereby reducing phosphorus absorption from the intestinal tract and lowering blood phosphorus levels (Shimada et al., 2004).

In a randomized controlled trial (Kawabata et al., 2022) comparing FDI and SFO in young Japanese women, the incidence of hypophosphatemia was 83.2 % in the SFO group, compared with 8.4 % in the FDI group. Concerning the duration of treatment, serum phosphorus levels in the SFO group did not fall below 2.0 mg/dL until after 4 weeks of treatment.

There have been several reports of hypophosphatemia caused by SFO, but there are few reports of hypophosphatemia for short treatment durations of 1 week or less.

Hypophosphatemia is considered to be particularly common in ICU (Geerse et al., 2010). The symptoms of hypophosphatemia are nonspecific, but severe cases are reported to involve encephalopathy, musculoskeletal disorders, dysphagia, and breathing problems. Mild hypophosphatemia has few symptoms, but chronic persistence is associated with muscle weakness and pain in the lower extremities and progressive osteomalacia. Causes of hypophosphatemia include, in addition to iron supplementation, decreased phosphorus absorption from the intestinal tract because of refeeding syndrome, inflammatory bowel disease, and other conditions; diarrhea; phosphorus transfer into cells due to respiratory alkalosis; and phosphate removal resulting from renal replacement therapy.

In this case, the patient's serum phosphorus level on admission was normal (4.3 mg/dL), and she had not been receiving any treatment or exhibiting any comorbidities prior to admission. Although the cause of iron deficiency anemia was unknown, it was suspected of being menstruation-related given that she was a young woman, and she was administered SFO. A decrease in the blood phosphorus level to <2.0 mg/dL was observed during the first 5 days of treatment, and high iFGF23 and iPTH levels and low 1,25-hydroxyvitamin D levels were observed during the first week of treatment. The reasons for the low 25-hydroxyvitamin D levels are unclear, but an association between low 25-hydroxyvitamin D levels and a higher risk of hypophosphatemia seems likely (Shimada et al., 2004).

The patient was not considered to have refeeding syndrome because there had been no episodes prior to admission suggesting abnormally low food intake. There was also no ongoing respiratory alkalosis, diarrhea, or vomiting. A number of drugs other than intravenous iron administration have been reported to cause hypophosphatemia. These include mannitol, carbonic anhydrase inhibitors, diuretics, and corticosteroids (Liamis et al., 2010). No diuretics were used in this case. Steroid pulse therapy was used to treat the anti-NMDA receptor encephalitis in this case and may also have contributed to the hypophosphatemia. The abovementioned resection of the ovarian teratoma as part of the treatment of anti-NMDA receptor encephalitis (Erlebah and Brandi, 2013), together with the steroid pulse therapy given concurrently with plasma exchange therapy conceivably could have resulted in decreased serum phosphate levels in this patient. However, hypophosphatemia attributable to glucocorticoid excess is not believed to be accompanied by an increase in FGF23 levels (Bosman et al., 2021). suggesting that the main cause of hypophosphatemia was SFO administration. The causes of hypophosphatemia are so varied that it is difficult to scrutinise them all, but the timing of SFO administration and the elevated iFGF23 levels make it likely that the main cause of hypophosphatemia in this case was due to the administration of SFO.

The JADER database, provided by the Pharmaceuticals and Medical Devices Agency in Japan, was used to identify cases of hypophosphatemia attributable to SFO administration reported between April 2004 and March 2023. The following parameters were used for the analysis.

Subjects: Cases submitted to JADER between April 2004 and March 2023. Target drug: saccharated ferric oxide. Adverse events: preferred terms “hypophosphataemia” or “hypophosphatemia” (PT code: 10021058, 10021059) and “blood phosphorus decreased” (PT code: 10049471) (MedDRA/J ver. 26.0, MedDRA, Japanese version).

In the search, 24 cases of hypophosphatemia suspected of being caused by SFO were reported, of which eight cases were registered with details about the dates of treatment initiation and adverse event onset (Table 3). Although detailed information is not available from the database, two cases were reported within 1 week after the start of SFO administration, including one case in a young woman in her 30s and the other in an elderly woman in her 80s. The doses were 40 or 80 mg, which did not deviate from standard dosages.

**Table 3:** Cases of hypophosphatemia suspected of being associated with SFO administration registered in JADER.

Age	Sex	Treatment start date	Date of adverse event	Treatment end date	Number of days to adverse event onset	Dose
80s	Female	20,060,928	20,061,003	20,061,012	5	40 mg
30s	Female	20,050,115	20,050,120	20,050,201	5	80 mg
50s	Male	20,050,521	20,050,606	20,050,613	16	80 mg
30s	Male	20,061,024	20,061,112	20,061,112	19	Unknown
40s	Female	20,080,112	20,080,202	20,080,202	21	80 mg
30s	Female	20,081,216	20,090,129	20,090,127	44	80 mg
20s	Female	20,171,001	20,171,010	20,171,023	9	Unknown
70s	male	20,190,208	20,190,312	20,190,303	32	80 mg

According to The National Database of Health Insurance Claims and Specific Health Checkups of Japan, during the year from April 2021 to March 2022, the total usage of FCM and SFO was 91,373 bottles and 5,194,191 ampoules, respectively. Although the information available makes it difficult to infer the number of patients using each of these drugs, it is clear that SFO and FCM are commonly used in Japan, even considering the fact that FCM is administered only once a week.

The current patient developed moderate hypophosphatemia (<2 mg/dL) on day 5 of SFO administration. On day 7, her iFGF23 and iPTH levels were elevated, and her blood 1,25-hydroxyvitamin D level was decreased. Our experience illustrates the importance of being aware of the possibility of hypophosphatemia from the early stage of SFO administration, regardless of the patient's age or dosage, and of monitoring the patient to prevent complications.

## Informed consent

Written consent was obtained from the patient's mother, as the patient herself had not fully recovered her comprehension.

## Ethical clearance

In consideration of the protection of the patient's personal information and privacy, consent was obtained in writing from the patient's mother.

## Funding source

This study has not received specific funding from any public, commercial, or non-profit sector funding agencies.

## CRediT authorship contribution statement

**Teruhisa Kinoshita:** Writing – original draft, Formal analysis. **Yuki Kondo:** Writing – original draft, Supervision, Project administration. **Yuka Sakazaki:** Validation, Formal analysis. **Hiroki Imaizumi:** Writing – original draft. **Norio Takimoto:** Supervision. **Yoichi Ishitsuka:** Supervision.

## Declaration of competing interest

The authors declare that they have no known competing financial interests or personal relationships that could have appeared to influence the work reported in this paper.

## Data Availability

No data was used for the research described in the article.

## References

[bb0005] Bosman A., van den Beld A.W., Feelders R.A., Zillikens M.C. (2021). Cortisol and phosphate homeostasis: Cushing’s syndrome is associated with reversible hypophosphatemia. Front. Endocrinol..

[bb0010] Camaschella C. (2015). Iron-deficiency anemia. N. Engl. J. Med..

[bb0015] Edmonston D., Wolf M. (2020). FGF23 at the crossroads of phosphate, iron economy and erythropoiesis. Nat. Rev. Nephrol..

[bb0020] Erlebah R., Brandi G. (2013). Effect and timing of operative treatment for teratoma associated N-methyl‑d-aspartate receptor-antibody encephalitis: a systematic review with meta-analysis. J. Neuroimmunol..

[bb0025] Geerse D.A., Bindels A.J., Kuiper M.A., Roos A.N., Spronk P.E., Schultz M.J. (2010). Treatment of hypophosphatemia in the intensive care unit: a review. Crit. Care.

[bb0030] Glaspy J.A., Wolf M., Strauss W.E. (2021). Intravenous Iron-induced hypophosphatemia: an emerging syndrome. Adv. Ther..

[bb0035] Hardy S., Vandemergel X. (2015). Intravenous iron administration and hypophosphatemia in clinical practice. Int. J. Rheumatol..

[bb0040] Ikuta K., Hanashi H., Hirai K., Ota Y., Matsuyama Y., Shimura A. (2019). Comparison of efficacy and safety between intravenous ferric carboxymaltose and saccharated ferric oxide in Japanese patients with iron-deficiency anemia due to hypermenorrhea: a multi-center, randomized, open-label noninferiority study. Int. J. Hematol..

[bb0045] Kawabata H., Tamura T., Tamai S., Fujibayashi A., Sugimura M., Study Group (2022). Intravenous ferric derisomaltose versus saccharated ferric oxide for iron deficiency anemia associated with menorrhagia: a randomized, open-label, active-controlled, noninferiority study. Int. J. Hematol..

[bb0050] Liamis G., Milionis H.J., Elisaf M. (2010). Medication-induced hypophosphatemia: a review. QJM Mon J Assoc Physicians..

[bb0055] Pasricha S.R., Tye-Din J., Muckenthaler M.U., Swinkels D.W. (2021). Iron deficiency. Lancet Lond Engl..

[bb0060] Shimada T., Hasegawa H., Yamazaki Y., Muto T., Hino R., Takeuchi Y. (2004). FGF-23 is a potent regulator of vitamin D metabolism and phosphate homeostasis. J. Bone Miner. Res. Off. J. Am. Soc. Bone Miner. Res..

[bb0065] Short M.W., Domagalski J.E. (2013). Iron deficiency anemia: evaluation and management. Am. Fam. Physician.

[bb0070] Vilaca T., Belmurugan N., Smith C., Abrahamsen B., Eastell R. (2022). Osteomalacia as a complication of intravenous Iron infusion: a systematic review of case reports. J. Bone Miner. Res..

[bb0075] Wolf M., Chertow G.M., Macdougall I.C., Kaper R., Krop J., Strauss W. (2018). Randomized trial of intravenous iron-induced hypophosphatemia. JCI Insight.

[bb0080] Wolf M., Rubin J., Achebe M., Econs M.J., Peacock M., Imel E.A. (2020). Effects of Iron Isomaltoside vs ferric Carboxymaltose on hypophosphatemia in Iron-deficiency Anemia: two randomized clinical trials. JAMA.

